# Cerebrospinal fluid levels of sortilin-1, lipocalin-2, autotaxin, decorin and interleukin-33 in patients with idiopathic intracranial hypertension

**DOI:** 10.1055/s-0042-1758559

**Published:** 2022-12-19

**Authors:** Ahmet Dündar, Adalet Arıkanoğlu, Hasan Hüseyin Özdemir, Hamza Aslanhan, Mehmet Uğur Çevik

**Affiliations:** 1 Mardin Artuklu University, Vocational School of Health Services, Department of Medical Laboratory, Mardin, Turkey.; 2 Dicle University, Faculty of Medicine, Department of Neurology, Diyarbakır, Turkey.; 3 A Hospital, Department of Neurology, İstanbul, Turkey.; 4 Dicle University, Faculty of Medicine, Department of Family Medicine, Diyarbakır, Turkey.

**Keywords:** Pseudotumor Cerebri, Lipocalin-2, Decorin, Interleukin-33, Sortilin-1, Autotaxin, Pseudotumor Cerebral, Lipocalina-2, Decorina, Interleucina-33, Sortilina-1, Autotaxina

## Abstract

**Background** Idiopathic intracranial hypertension (IIH) is characterized by increased cerebrospinal fluid (CSF) pressure of unknown cause. It has been suggested that the inflammatory process plays a role in the pathophysiology of the disease. Sortilin-1, lipocalin-2, autotaxin, decorin, and interleukin-33 (IL-33) are among the factors involved in inflammatory processes.

**Objective** To investigate the CSF levels of sortilin-1, lipocalin-2, autotaxin, decorin, and IL-33 in patients with IIH.

**Methods** A total of 24 IIH patients and 21 healthy controls were included in the study. Demographic characteristics of the patients and of the control group as well as CSF pressures were evaluated. Sortilin-1, lipocalin-2, autotaxin, decorin and IL-33 levels in the CSF were measured.

**Results** The CSF levels lipocalin-2, sortilin-1, autotaxin, IL-33 and CSF pressure were significantly higher in the patients group compared with the control group ( *p* < 0.001). Decorin levels were reduced in patients ( *p* < 0.05). There was no correlation between the autotaxin and IL-33 levels and age, gender, CSF pressure, and body mass index. The results of our study showed that inflammatory activation plays an important role in the development of the pathophysiology of IIH. In addition, the fact that the markers used in our study have never been studied in the etiopathogenesis of IIH is important in explaining the molecular mechanism of this disease.

**Conclusion** Studies are needed to evaluate the role of these cytokines in the pathophysiology of the disease. It is necessary to evaluate the effects of these molecules on this process.

## Resumo

**Antecedentes** A hipertensão intracraniana idiopática (HII) é caracterizada pelo aumento da pressão do líquido cefalorraquidiano (LCR) de causa desconhecida. Tem sido sugerido que o processo inflamatório desempenha um papel na fisiopatologia da doença. Sortilina-1, lipocalina-2, autotaxina, decorina e interleucina-33 (IL-33) estão entre os fatores envolvidos nos processos inflamatórios.

**Objetivo** Investigar os níveis de sortilina-1, lipocalina-2, autotaxina, decorina e IL-33 no LCR de pacientes com HII.

**Métodos** Um total de 24 pacientes com HII e 21 controles saudáveis foram incluídos no estudo. Foram avaliadas as características demográficas dos pacientes e do grupo controle, bem como as pressões liquóricas. Os níveis de sortilina-1, lipocalina-2, autotaxina, decorina e IL-33 no LCR foram medidos.

**Resultados** Os níveis no líquido cefalorraquidiano lipocalina-2, sortilina-1, autotaxina, IL-33 e pressão liquórica foram significativamente maiores no grupo de pacientes em comparação com o grupo controle (p < 0,001). Os níveis de decorina foram reduzidos nos pacientes (p < 0,05). Não houve correlação entre os níveis de autotaxina e IL-33 e idade, sexo, pressão liquórica e índice de massa corporal. Os resultados do nosso estudo mostraram que a ativação inflamatória desempenha um papel importante no desenvolvimento da fisiopatologia da HII. Além disso, o fato de os marcadores utilizados em nosso estudo nunca terem sido estudados na etiopatogenia da HII é importante para explicar o mecanismo molecular dessa doença.

**Conclusão** Estudos são necessários para avaliar o papel dessas citocinas na fisiopatologia da doença. É necessário avaliar os efeitos dessas moléculas nesse processo

## INTRODUCTION

Idiopathic intracranial hypertension (IIH) is one of the rare diseases characterized by increased CSF pressure. The disease particularly affects the young population. There is a high rate of headache and vision loss is observed in ∼ 25% of patients. 1 Obesity is the most important predisposing factor, and the disease is observed especially in overweight women in their productive period. The etiopathogenesis of the disease has not been fully determined. It has been suggested that some cytokines and steroid hormones secreted from adipocyte tissue may affect CSF production and drainage. The effects of obesity-related chronic inflammation on the development of the disease have been evaluated in recent years. Proinflammatory cytokines such as leptin, IL-2, 17, macrophage chemotactic protein-1 and plasminogen activator inhibitor-1 were significantly increased in patients with IIH. 2 3 4 5 6 7

Sortilin-1 is a 95 kDa transmembrane glycoprotein. Its role in many neurological and hematological diseases has been evaluated. 8 9 Autotaxin is a secreted enzyme important for generating the lipid signaling moleculely so lisophosphatidic acid (LPA). Autotaxin has lysophospholipase D activity that converts lysophosphatidyl choline into LPA. Lysophosphatidic acid evokes growth factor-like responses including stimulation of cell proliferation and chemotaxis. 10 11 Decorin is a prototype member of the small leucine-rich proteoglycan (SLRP) and it has been observed to play a role in inflammation, fibrotic disorders, and cancer. 12 Lipocalin-2 (LCN2) is secreted from many cell types and can be considered as a biomarker of inflammation ischemia and infection. 13 IL-33 is a member of the IL-1 super family and protein encoded by the IL-33 gene in humans. 14 It is expressed in epithelial, endothelial, and fibroblast-like cells during homeostasis. Also, it has been shown to be associated with allergic, fibrotic, and chronic inflammatory conditions. 15

In our literature review, there was no study evaluating the role of these molecules in the physiopathology of IIH disease. Hence, the aim of the present study was to investigate the role of inflammation in the pathogenesis of IIH using these molecules.

## METHODS

The patients were included among the patients who applied to the neurology outpatient clinic of Dicle University. The present study was approved by the ethics committee of Dicle University (2016/156) and written informed consent was obtained from all participants prior to their inclusion into the study. Routine biochemical tests, magnetic resonance imaging (MRI) of the brain and MRI cerebral venography were performed in the patients. A detailed eye examination and visual field evaluation were performed for each patient. Disease duration, complaints, symptoms, and examination findings were recorded. Patients were diagnosed with IIH according to the Dandy criteria. 16

The control group consisted of healthy volunteers who applied to the check-up outpatient clinic and did not have any complaints. A detailed neurology and physical examination was performed in this group. The fundus and visual field were evaluated. The body mass index (BMI) of the patient and control groups was determined. It was calculated as weight in kg/height in m ^2^ . It was noticed that the control group was similar to the patient group in terms of age, gender and BMI.

Those with a history of autoimmune disease, cardiac and thromboembolic disease, and diseases that cause secondary IIH (sinus thrombosis, intracranial mass, high-dose vitamin A or steroid therapy) were not included in the study. In addition, samples with findings indicating other diseases in biochemical examinations in the CSF taken were not included in the study.

### Biochemical analyses

All participants in the study were informed about the study prior to consenting to participate. Lumbar CSF samples were withdrawn into tubes. Cerebrospinal fluid was centrifuged at 400 g for 10 minutes at 10°C. The supernatant was collected. In accordance with the protocol of the manufacturer, CSF samples were taken from the patient and control groups and then kept in the laboratory. The CSF samples were transferred on ice and stored at - 80 ^°^ C until the end of the study. The levels of lipocalin-2, sortilin-1, decorin, IL-33 and autotaxin were measured using commercially available enzyme-linked immunosorbent assay kits (YLbiont, Kit LTD, China). The absorbance was 450 nm and recorded by an absorbance microtiter plate reader (ELx800TM, BIO-TEK instruments, USA).

### Statistical analysis

Statistical analyses were performed using PASW Statistics for Windows, version 18.0 (SPSS Inc., Chicago, IL, USA). The categorical variables were expressed as numbers and percentages. The Mann-Whitney U test was applied to compare the differences between the two independent groups when the dependent variable was either ordinal or continuous. The Kruskal-Wallis test was implemented to compare more than two groups. Spearman correlation analysis was employed to determine the relationships between the data. The level of statistical significance was set at 5%.

## RESULTS

Twenty-four patients with IIH and 21 healthy controls were enrolled in the present study. The mean age of the patient group (14 females and 10 males) was 31.50 years old, while the mean age of the control group (10 females and 11 males) was 32.00 years old. The clinical features and MRI findings of the patients are given in Table 1 There was no statistically significant difference between the two groups in terms of sex ( Table 2 ).

**Table 1:** Clinical signs of patients

		*n*	%
Clinical symptoms	Headache	23	56.09
	Tinnitus	12	29.26
	Diplopia	6	14.63
Ophthalmic features	Bilateral papilledema	23	76,66
	Unilateral papilledema	1	3.33
	Sixth-nerve palsy	6	20.0
MRI Findings	Empty sella turcica	8	47.05
	Optic nerve protrusion	3	17.64
	Optic nerve tortuosity	3	17.64
	Posterior globe flattening	3	17.64

Abbreviation: MRI, magnetic resonance imaging.

**Table 2:** Demographic values of the idiopathic intracranial hypertension group and control group

	**Control group** **(** ***n*** ** = 21)**	**Idiopathic intracranial hypertension group** **(** ***n*** ** = 24)**	***p-value***
Sex	Female	10 (47.6%)		14 (58.3%)
	Male	11 (52.4%)		10 (41.7%)
Age	32.00 (20.00–45.00)	31.50 (20.00–45.00)	0.945
BMI	26.00 (23.00–29.00)	26.50 (22.00–29.00)	0.350
Cerebrospinal fluid pressure	18.00 (14.00–22.00)	31.00 (27.00–40.00)	< 0.001

Abbreviation: BMI, body mass index.

Note: Data are expressed as median (minimum–maximum); the degree of

**Figure 1.**
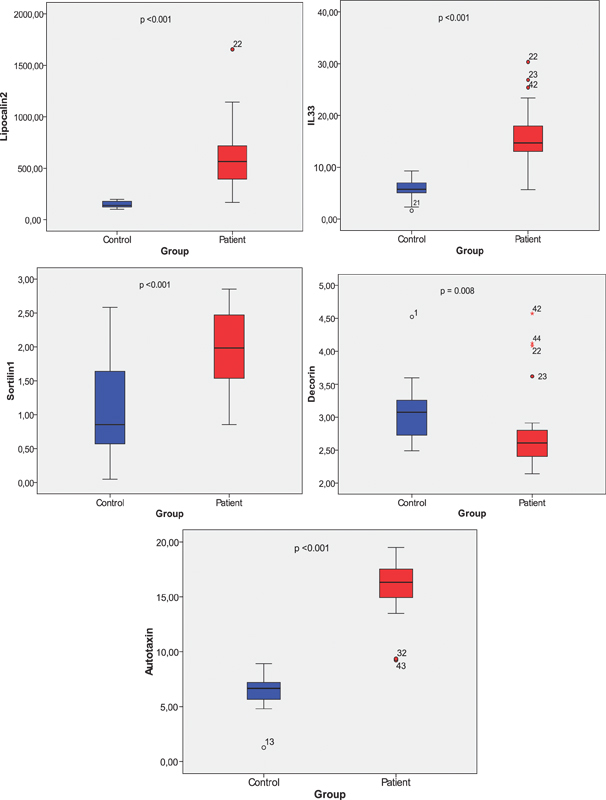
Lipocalin-2, sortilin-1, decorin, IL-33 and autotaxin levels in the cerebrospinal fluid of idiopathic intracranial hypertension patients and controls.

significance of comparison between the patient and control group was set at *p* < 0.001,

The cerebrospinal fluid pressure in the patients with IIH and healthy individuals were 31.00 and 18.00 respectively. The CSF levels of lipocalin-2 in patients with IIH and in healthy individuals were 564.21 pg/ml and 138.75 pg/ml, respectively. The CSF levels of sortilin-1 in patients with IIH and in healthy individuals were 1.98 pg/ml and 0.85 pg/ml, respectively. The CSF levels of IL-33 in patients with IIH and in healthy individuals were 14.71 pg/ml, and 5.75 pg/ml, respectively. The CSF levels of autotaxin in patients with IIH and in healthy individuals were 16.33 pg/ml, and 6.65 pg/ml, respectively. The CSF levels of lipocalin-2, sortilin-1, interleukin-33, autotaxin, and CSF pressure were significantly increased in patients with IIH compared with those in the control group ( *p* < 0.001). The CSF levels of decorin in patients with IIH and in healthy individuals were 2.61 pg/ml, and 3.07 pg/ml, respectively. Cerebrospinal fluid levels of decorin were decreased in patients with IIH compared with those in the control group ( *p* < 0.05) ( Table 3 , Figure 1 ).

**Table 3:** Lipocalin-2, sortilin-1, decorin, IL-33, and autotaxin levels in the cerebrospinal fluid of ıdiopathic ıntracranial hypertension patients and control group

Parameters	Control group	Idiopathic intracranial hypertension group	*p* - *value*
Lipocalin-2 (pg/ml)	138.75 (101.34–197.87)	564.21* (169.07–1054.86)	< 0.001
Sortilin-1 (pg/ml)	0.85 (0.05–2.59)	1.98 * (0.85–2.85)	< 0.001
Decorin (pg/ml)	3.07 (2.49–4.52)	2.61 ** (2.14–4.57)	0.008
Interleukin-33 (pg/ml)	5.75 (1.62–9.31)	14.71 * (5.69–30.34)	< 0.001
Autotaxin (pg/ml)	6.65 (1.26–8.90)	16.33 * (9.24–19.51)	< 0.001

Notes: Data are expressed as median (minimum–maximum); ^*^ *p* < 0.001; ^**^ The degree of significance of comparison between the patient and control groups was set at *p* < 0.05.

The levels of sortilin-1 and decorin in patients with IIH were positively correlated with their IL-33 levels ( *p* < 0.01). The mean CSF pressure levels in the patients with IIH were positively correlated with their age. The levels of lipocalin-2 in patients with IIH were positively correlated with their age and autotaxin levels ( *p* < 0.05). No correlation was detected between these molecules and BMI in any of the groups.

## DISCUSSION

In the present study, we first found increased CSF levels of lipocalin-2, sortilin-1, autotaxin, and IL-33 in patients with IIH. On the other hand, CSF decorin levels were found to be decreased in patients with IIH. Also, no correlation of these molecules with BMI and CSF pressure was observed in the patient and control groups.

The mechanism of these molecules in the etiopathogenesis of IIH has not been clarified yet, since there are no studies on CSF or serum levels of sortilin-1, lipocalin-2, autotaxin, decorin and IL-33 in patients with IIH. Therefore, our study is the first clinical study in the literature.

Sortilin-1 is a proinflammatory cytokine secreted particularly in the hippocampus, the dentate gyrus and the cerebral cortex. 17 In vivo studies suggests that sortilin plays a significant role in the pathogenesis of vascular, metabolic, and inflammatory disorders. 18 It has been determined that its serum levels increase in inflammatory diseases and it can be considered as a proinflammatory molecule. 19 Serum sortilin-1 levels were evaluated in some neuropsychiatric diseases. In particular, sortilin-1 plays a role in the pathophysiology of Alzheimer and some types of dementia. 20 It was found that the serum level increased in patients with depression and showed a positive correlation with age and BMI. 21 In our study, we found that levels of CSF sortilin-1 were significantly higher in the patient group. There is evidence that the sortilin-1 molecule has a role in the intracellular cytokine cascade and platelet activation. 22 The high levels of CSF sortilin-1 in patients with IIH probably suggest that sortilin-1 facilitates platelet activation and therefore increases CSF drainage. This suggests that platelet activation also increases cytokine release.

Autotaxin is widely expressed in the brain, the spinal cord, the ovaries, the lungs, the intestines, the kidneys, and in lymph nodes and has been shown in many inflammatory diseases such as idiopathic pulmonary fibrosis, rheumatoid arthritis, and chronic interstitial lung diseases. 23 Local or systemic infections increase autotaxin and LPA levels. 24 Dysfunctional expression and activity of autotaxin with associated changes in LPA signaling is implicated in the pathogenesis of Alzheimer disease and autotaxin level was found to be high in Mild Cognitive Impairment (MCI), and Alzheimer's disease (AD) patients in the studies performed. 10 25 In our study, we found the CSF autotaxin level to be high. The mechanism of the autotaxin molecule in patients with IIH indicates that H _2_ O _2_ may be increased probably due to inflammation in the adipose tissue, and that the increase in the autotaxin level may be for the purpose of protecting microglial cells. 26 In addition, the cytokines released by the autotaxin product LPA cause the autotaxin molecule to be oversynthesized. The increase in autotaxin also causes an increase in LPA again, thus a continuous positive inflammatory cycle occurs. 27

Extracellular decorin is the structural component of the matrix and it mediates cellular collagen fibrillogenesis, wound healing, inflammation, and neovascularisation. 28 Decorin has been reported to have a neuroprotective effect in some studies. Levels have been shown to decrease significantly in chronic diseases with inflammation. 29 Decorin infusion prevented brain damage by showing an anti-inflammatory effect in rats with hydrocephalus. 30 Özay et al. showed that decorin inactivates transforming growth factor β1 and protects the brain tissue and neuronal cells after traumatic brain injury. 31 Intracranial hypertension is also a disease in which neuronal damage and inflammation are clearly observed, and we found that CSF decorin level was significantly low in the patient group.

Lipocalin-2 is a family of secreted adipokines that play important roles in acute and chronic inflammation. It is a proinflammatory molecule that takes an active role especially after CNS inflammation, damage, and infection. 32 It has been shown to play an effective role in experimental autoimmune encephalomyelitis models and in the etiopathogenesis of multiple sclerosis. In addition, CSF levels are increased in patients with progressive multiple sclerosis. 33 Oxidative stress is defined as an imbalance between free oxygen radicals and the antioxidant system. 34 Studies have suggested that conditions such as epilepsy, Alzheimer disease, multiple sclerosis, and spinal cord disease may be associated with oxidative stress. 35 Ischemia has been observed to be associated with increased free oxygen radicals in reperfusion injury, heart attack, stroke and increased lipocalin-2. It was stated that an increase in lipocalin-2 synthesis may be in free oxygen radicals. 36 In our study, however, CSF lipocalin-2 molecule was found to be significantly higher than the control group. The increase in levels of lipocalin-2 in patients with IIH indicates that the oxidant and antioxidant balance may be impaired, which may increase the levels of lipocalin-2, which acts as an acute phase reactant, as a result of increased free oxygen radicals.

Interleukin-33 participates in Th2-associated immune reactions. 37 It is expressed in specific regions of the brain and the spinal cord and mediates the interaction between immune, endothelial and CNS resident cells. 38 Interleukin-33 is activate inflammatory cells, including glial cells also it is involved in the neuroinflammation of many neurological diseases such as AD and MS. It has also been determined that IL-33 can be used as a biomarker that determines the damage in diseases such as ischemic stroke, in which parenchymal damage is observed. 39 In our study, we found that CSF IL-33 levels were significantly increased. It indicates that increased CSF IL-33 levels may be an indicator of inflammation.

Different results have been obtained in many studies related to the correlation between BMI, CSF pressure, and biochemical parameters in IIH patients. EI-Tamawy et al. 40 showed that, in IIH patients, no correlation was observed between BMI and cytokine levels such as IL-4, IL-10, and TNF-α. In another study conducted with IIH patients, no significant correlation was found between CSF/serum leptin levels, BMI, and CSF pressure. 6 In our study, no correlation was observed between BMI, CSF pressure, and biomarkers.

The present study has several limitations. The fact that these biomarkers were studied only in CSF fluid in our study and not in serum samples is among the main limitations of our study. Another limitation of our study is that the biochemical properties were not included in the study.

In conclusion, in our study, it was determined that lipocalin-2, sortilin-1, autotaxin, IL-33 and CSF pressure levels increased and decorin levels decreased in the levels CSF. The results of our study showed that inflammatory activation plays an important role in the development of the pathophysiology of IIH. In addition, the fact that the markers used in our study have never been studied in the etiopathogenesis of IIH is important in explaining the molecular mechanism of this disease. Studies are needed to evaluate the role of these cytokines in the pathophysiology of the disease.
